# Atlanta Academy of Medicine

**Published:** 1875-12

**Authors:** R. W. Westmoreland

**Affiliations:** Reporter


### Atlanta Academy of Medicine.

R. W. WESTMORELAND, M.D., REPORTER.

ATLANTA, GA., October 5, 1875.

Academy met, Vice-President, Dr. Rauschenburg in the chair.

Report of cases being in order, Dr. Baird read a communication from Dr. C. Drew, Jacksonville, Florida, corresponding member of the Academy, relating to cases of pernicious intermittent fever occurring among the butchers more particularly. The butchers having to go before day to carry their meat to market, and then, after working till late in the morning, go home through the sun to breakfast, the doctor supposed that the malarial poison was more readily absorbed on account of their fatigued condition—they having to go home through a malarious district.

Dr. Baird asked to know if, in the experience of any member, butchers especially had been subject to such attacks in this city.

No member recollected any such tendency in this locality.

Dr. J. G. Westmoreland thought the principal point in the report was the time the malaria was inhaled. It is generally believed that malaria is not inhaled after the sun rises to any height, and therefore he thought that the butchers, having to go some distance before day through a malarious district, were more particularly subject to the influence.

Dr. J. G. Westmoreland reported a case of poisoning by opium, in which the subject had taken half a fluid ounce of laudanum twenty minutes before he saw him, and perhaps had taken as much more previously, from the history of the case. Found him in a state of stupor, but with considerable effort was aroused sufficiently to be induced to swallow some warm water. By the stomach tube and vomiting of the patient, the stomach was relieved of what had not been absorbed. Repeated hypodermic injections of atropia for half an hour, by which one-fifth of a grain was used, changed the pupil from very great contraction to considerable dilation, after which the patient revived from his stupor. When last seen, had symptoms resembling intoxication. Pulse, when first seen, was about fifty, left him with a pulse of seventy-five or eighty.

Dr. Armstrong reported three cases of typhoid fever, commencing with remissions, which led him astray as to the true character of the fever for the first few days. Desired to know the experience of members as to the occurrence of intermission in the commencing of typhoid fever. All the cases had pneumonic symptoms, which proved to be the greatest difficulty in treatment.

Dr. J. G. Westmoreland desired to know the duration of the disease in the cases reported.

Dr. Armstrong stated that the first died after twenty days; the second after thirty days, with hemorrhage from the lungs; and the third, now under treatment, now eighteen days from commencement, is doing very well.

Dr. J. G. Westmoreland stated that the opinion once prevailed that typhoid fever was invariably continued in its character, but it has been ascertained that it is sometimes remittent and occasionally intermittent in the commencement of the disease. Remembers the first case he ever treated was entirely intermittent for several days at the outset; has seen several since of like character. Some have used quinine to test the character of the fever where remissions existed.

Dr. Baird reported a case of remittent fever, for which quinine was prescribed. It failed to arrest the fever, and subsequent developments proved it to be typhoid fever.

Dr. W. F. Westmoreland was called to perform the operation of œsophagotomy for supposed chicken bone in œsophagus. The attending physician thought that he felt distinctly the bone, and tried to extract it but failed. Found the parts excessively swollen—so much so that it was impossible to swallow solids. Pulse one hundred and ten to one hundred and fifteen, with heat of skin. The operation of œsophagotomy being decided on, the difficulty was to locate the point where the incision should be made, as the tumefaction was so great that the usual guides were entirely obscured. Left side of neck was selected, and, fortunately, the incision was made at the right point. The incision having been made to the œsophagus, considerable pus was discharged, but the œsophagus being opened no bone was discovered, either above or below the opening. No vessel was injured requiring ligature. Did not know what had become of the bone; desired to know the opinion of others as to propriety of operation. The œsophagus had softened considerably, and was easily torn. There seemed to be no great shock to the patient at the time of operation, but has learned that he died 48

hours after the operation. Did not follow the plan of Dr. Gross in closing the œsophagus and skin with sutures, but preferred and followed that of Dr. Chiver in leaving the opening free. Dr. C. performed three operations in this manner, two of which recovered.

A record of twenty-five operations of œsophagotomy shows a very favorable per cent. of recovery.

Academy adjourned.

---

ATLANTA, October 19, 1875.

Academy was called to order by the President, Dr. John M. Johnson.

Dr. Calhoun reported four cases, in which enucleation of eyeball was performed. He exhibited to the Academy the preserved specimens, and remarked that the injury to the eye resulted each from different causes. The first case reported was that of a man aged twenty-one years, who received a cut in the right eye, resulting in total destruction of vision. The instrument by which the wound was given entered at the lower corneo-sclerotic junction, piercing the ciliary bodies. Four weeks after the reception of the injury, sympathetic inflammation was set up in the left eye, in which the vision began to fail with great pain. The consent of the patient to an operation having been refused until five months afterwards, when he was induced to submit, and the operation of enucleation was performed. The patient was kept in bed four or five days, and in that time the inflammation subsided, and vision was perfectly restored in the left or sound eye.

The second case was that of an old man aged fifty, who presented himself five weeks ago. Two years ago contracted syphilis. Six months afterward had syphilitic iritis, with gummy tumors upon the iris of the right eye. Destruction of vision supervened, which was not removed by curing the disease, but the ball shrivelled up, and became indurated. Six months ago a slight irritation was set up in the left eye, with gradual failure of vision, and increase of irritation and pain in both. General health failing, and consent to operation being obtained, the ball of the right eye was extracted by enucleation. Treated in the usual manner, and the recovery was complete—sight in the left eye restored.

The third case was a woman sixty years old, who presented herself four weeks ago, with left eyeball immensely inflamed, red and swollen, protruding considerably from the eyelids. Sympathetic inflammation involved the right eye, and enucleation was resorted to, with the recovery of the sound eye. This was evidently a case of *ideopathic* irido-choroiditis, as no cause could be found to which it was attributable. Some supposition of syphilitic origin obtained, but a thorough examination dispelled any such.

The fourth case reported was that of a man thirty-three years old, who two weeks ago, contracted gonorrhœa, from which he had gonorrhœal ophthalmia in the left eye. The attending physicians resorted to the usual means to suppress the gonorrhœal ophthalmic inflammation without success. The cornea sloughed off, permitting the protrusion of the contents of the ball. When presented to Dr. Calhoun, the right eye began to show signs of sympathetic inflammation, and increased so rapidly that enucleation was deemed necessary.

He desired to call attention to the fact that in the four cases there was a sympathetic inflammation produced in each by different causes, namely: traumatic, idopathic, syphilitic and gonorrhœal.

Dr. Logan called attention to the apparent antidotal effect of veratrum manifested in the recent use by him of this drug in an acute attack of disease, in which narcotism had been produced by the injudicious use of opium. He administered veratum to allay febrile symptoms with relief of the stupor resulting.

Dr. Todd mentioned a report he made in the American Journal of Medical Sciences, of November, 1872, of a case of opium poisoning, which recovered on the use of veratrum. In compliance with the request that he report other cases in which he had used the remedy, he reported, first, the case of a baker, who took 2℥ of tinct. opii. He was bent on suicide, refused to take any remedy, but by considerable effort was induced to swallow thirty grains of sulphate of zinc, and sixty grains of ipecac, with repetition of zinc without vomiting. Notwithstanding this, the patient grew rapidly worse, and the cold douche mustard and electricity were resorted to. An hour afterward one-fourth of a grain of atropia was administered. Shortly after this the pulse was fifty, respiration ten, with pupils greatly dilated, the pulse growing weaker, and coma persistent. Pulse fell as low as forty, when it occurred to him to give veratrum. Three drops were given hypodermically, repeated in ten to fifteen minutes, after which the patient became conscious. In fifteen minutes gave another injection of three drops, which produced recovery complete.

The second case, a man forty-five years old, who had taken 1℥ of gum opium an hour before. When seen he was highly intoxicated, with profuse perspiration. Persistently refused to take any remedy. A male catheter was introduced into the nose, and down the throat, through which sixty or seventy grains of sulphate of zinc and as much ipecac was passed into the stomach. Immediately great quantities of opium were vomited. He was continually worried and walked, yet the comatose condition increased. Ten to twelve drops of veratrum were given hypodermically every half hour, producing emesis at each time. For eight hours after no great contraction of pupils, nor any considerable degree of unconsciousness. At the expiration of eight hours the family physician was called in, and only one more dose of veratrum was given. Pulse was sixty-five, respiration fifteen or sixteen. Cold douche and atrophia were resorted to an hour after, until the pupils were dilated, they having contracted for an hour or so, but with perceptible increase of stupor. Mustard was applied to the spine and extremities. Ten hours after he was first seen he died of asphyxia, the heart continuing to act for several minutes after breathing had stopped. An attempt to re-establish breathing failed.

The next was a child ten years old, affected with meningitis, to which, after being bled, an over dose of morphine was given by the attending physician. When called in consultation, the patient showed all the symptoms of opium poisoning, and in half an hour was in comatose condition. Three drops of veratrum were injected hypodermically until nine drops were used. Twelve hours after the child died, with widely dilated pupils, from the force of the disease.

He remarked that in case of poisoning, whether from opium or alcohol, he thought an emetic did good not only by relieving the stomach of what poison it might contain, but also eliminated the poison from the system. Call the effect revulsion, or what not, the result is the same. Reported some experiments he had heard of by Dr. Reese with atropia on dogs, in which large doses did not do so much damage as smaller ones combined with opium.

Dr. Baird made a motion that Dr. Logan be requested to read to the Academy his report on the suppression of small-pox, prepared for the State Board of Health, which was adopted.

Dr. Logan then read a paper, in which he insisted upon the necessity of having means adopted by which vaccination would be more thoroughly attend to. He spoke of the theory that other diseases might be transmitted through the medium of the vaccine virus, and desired to know the disease in which the evidence was conclusive. Wished the opinion of members.

Dr. Miller stated that his experience was negative, never having witnessed any thing of the kind. Thought, however, that there should be great caution exercised, as there was a probability of such an occurrence.

Academy adjourned.

---

ATLANTA, GA., October 26, 1875.

Academy called to order by the Vice-President, Dr. V. H. Taliaferro.

Report of cases:

Dr. Woodhull mentioned a case of threatened miscarriage which he had treated six or eight weeks with viburnum prunifolium. This case was six months advanced in pregnancy, and he was satisfied from the nature of the pains that they were from uterine contractions, although the os uteri had not dilated. Gave the remedy in ℨj of flu. extract. Only recently has his attention been called to the sedative action of this remedy on the uterus, and desired the experience of members as to its efficacy.

Dr. Armstrong replied that he had used the remedy often in conjunction with opium in the dose of from one dessertspoonful to a tablespoonful, repeated in one, two, and three hours, according to the nature of the case.

Dr. Taliaferro had used it in several cases, with only one failure to control the contractions. In this case the woman was six months advanced when he was called in, and found the pains very active. Administered viburnum in fℨj doses, with x. to xv. grs. brom. potass., which quieted the uterus. He left directions to repeat the dose at certain intervals, should the pains continue to recur. By failure to comply properly with instructions, pains came on again with renewed activity. Again the remedy quieted the uterus. For three or four days there was no sign of returning, when, without premonition, pains came on and the membranes ruptured. Remedy used then with former result, but two days after she miscarried with twins. In one the membrane was ruptured, the other was not. The second case reported was a woman in the habit of aborting at about the sixth month period. Viburnum and brom. potass, were prescribed two or three times a day until pains coming on, it was given every two or three hours. The woman went to full term.

Dr. Armstrong reported a case of evident scarlet fever. Mentioned all the characteristic symptoms of this disease as being plainly manifested in this case.

Dr. Logan having seen the case with Dr. Armstrong, unhesitatingly confirmed his diagnosis. Mentioned, as a point overlooked by Dr. Armstrong in his report, the mulberry tongue which was very marked in this case.

Dr. R. W. Westmoreland reported a case of supposed premature rupture of membrane in which, three or four days before labor, the patient had a sudden discharge of water from the vagina, unattended with pain. At the time of the labor neither he nor the patient observed any discharge of water. Desired to have the opinion of members as to the probability of rupture of membrane in this case.

Dr. W. F. Westmoreland desired to know if any member had discovered diptheria in the city. His attention had been called to its presence. Had not seen any himself.

Dr. Logan had seen one case of distinctly marked diptheria, with recovery.

Dr. W. F. Westmoreland reported a case of poisoning by ext. nux vomica. A lady having taken about eight times as much as the prescribed dose, seven hours after had all the symptoms of strychnine poisoning. Prompt relief was given by the use of chloroform and ether by inhalation. Ether was discontinued on account of its suffocating effects. For three hours the patient was slightly under the influence of the chloroform. Great nervousness and soreness were the after symptoms, which were allayed by eight grains of chloral, and recovery was complete.

Dr. Baird desired to know in what manner chloroform proved curative or antidotal.

Dr. W. F. Westmoreland thought it was not antidotal, but quiets the excitement through the nervous system. As strychnine destroys life in a manner similar to tetanus by excessive muscular action, and consequent exhaustion, chloroform controls this by its anæsthetic effect upon the nervous system.

Dr. Woodhull thought that the excessive irritation of the spine by the strychnine was counteracted by the soothing effect of the chloroform. The fact that dogs die suddenly from strychnine denys the assertion that death by exhaustion is the usual result.

Dr. W. F. Westmoreland remarked that very large doses, by paralyzing the power of respiration, through excessive nervous action, produced sudden death.

Dr. Taliaferro thought chloral possessed antidotal powers in strychnine poisoning. Mentioned the occasion of its use in the poisoning of a favorite dog by strychnine. Twenty to twenty-five grains of chloral, repeated in an hour, were forced down the throat. In two hours the spasms ceased, and the dog was seemingly quite well. Supposed the action of chloral similar to chloroform in such poisoning.

Dr. Baird then understood that strychnine, producing a local irritation, was soothed or neutralized by the anæsthetic action of chloroform upon the nervous centres.

Dr. W. F. Westmoreland explained that his idea was that each action had its effect through the nervous system—the one exciting, the other soothing.

Dr. Armstrong remarked that he had seen but one case of strychnine poisoning, and this he attributed to an over dose of Collin's antidote, by a gentleman in the habit of using opium. This preparation contains, as he believes, strychnine.

Dr. Logan reported a case in which he was informed by the lady's husband that the antidote has about the same effect as the opium, her appetite being as good for the antidote as for the opium. Hence he believed that opium was the principal ingredient.

Dr. Todd remarked that he believed opium constituted the principal ingredient, merely in a colored form. Does not think that strychnine enters into the composition.

Academy adjourned.

